# Interfering Role of ERα on Adiponectin Action in Breast Cancer

**DOI:** 10.3389/fendo.2020.00066

**Published:** 2020-02-18

**Authors:** Giuseppina Daniela Naimo, Luca Gelsomino, Stefania Catalano, Loredana Mauro, Sebastiano Andò

**Affiliations:** ^1^Department of Pharmacy, Health and Nutritional Sciences, University of Calabria, Arcavacata, Italy; ^2^Health Center, University of Calabria, Arcavacata, Italy

**Keywords:** breast cancer, obesity, adiponectin, estrogen receptor alpha, adipose tissue

## Abstract

Obesity is characterized by an excess of adipose tissue, due to adipocyte hypertrophy and hyperplasia. Adipose tissue is an endocrine organ producing many bioactive molecules, called adipokines. During obesity, dysfunctional adipocytes alter adipokine secretion, contributing to pathophysiology of obesity-associated diseases, including metabolic syndrome, type 2-diabetes, cardiovascular diseases and many types of malignancies. Circulating adiponectin levels are inversely correlated with BMI, thus adiponectin concentrations are lower in obese than normal-weight subjects. Many clinical investigations highlight that low adiponectin levels represent a serious risk factor in breast carcinogenesis, and are associated with the development of more aggressive phenotype. A large-scale meta-analysis suggests that BMI was positively associated with breast cancer mortality in women with ERα-positive disease, regardless menopausal status. This suggests the importance of estrogen signaling contribution in breast tumorigenesis of obese patients. It has been largely demonstrated that adiponectin exerts a protective role in ERα-negative cells, promoting anti-proliferative and pro-apoptotic effects, while controversial data have been reported in ERα-positive cells. Indeed, emerging data provide evidences that adiponectin in obese patients behave as growth factor in ERα-positive breast cancer cells. This addresses how ERα signaling interference may enhance the potential inhibitory threshold of adiponectin in ERα-positive cells. Thus, we may reasonably speculate that the relatively low adiponectin concentrations could be still not adequate to elicit, in ERα-positive breast cancer cells, the same inhibitory effects observed in ERα-negative cells. In the present review we will focus on the molecular mechanisms through which adiponectin affects breast cancer cell behavior in relationship to ERα expression.

## Introduction

Overweight and obesity are characterized by increased fat tissue accumulation. The World Health Organization classified as overweight people, subjects with body mass index (BMI) > 25 kg/m^2^, and as obese, people with BMI > 30 kg/m^2^ (30.0–34.9, grade I; 35.0–39.9, grade II; and 40, grade III). These pathological conditions are known to have a global impact on public health and are getting substantial attention worldwide, being recognized as the epidemic of the twenty-first century. Overweight/obese patients have nearly doubled since 1980s and nowadays represent a third of the world population and by the year 2030 will reach the 57.8% (1, 2). Specifically, the prevalence of overweight individuals was 38.5% in men and 39.4% in women, and the proportion of obese patients was 10.1% in men and 14.8% in women. These data underline a higher incidence in women (3). Moreover, obesity is considered a comorbid condition, determining a severe health public concern since it is associated with several diseases, such as diabetes, coronary artery disorders, hypertension, kidney illness, disability and poor mental health deeply negatively impacting quality of life, work productivity and healthcare expenses (4–7). In addition, several clinical trials described a correlation between excess of body weight and the development and progression of many cancers, especially in post-menopausal breast tumors (8–17). Nevertheless, most studies evidenced that the effect of obesity on pre-menopausal or post-menopausal breast cancer risk are closely related to the disease subtypes, exhibiting ERα expression (9). However, obesity is associated with to poor breast cancer outcomes irrespective of menopausal status (9).

Breast carcinoma is a complex and heterogeneous disease, whose development and progression depends on various genetic predispositions and several established factors (age, sex, family history, gene mutations, reproductive factors). It is now clear that modifiable factors, such as alcohol drinking, smoking, physical inactivity and overweight, also have an important role in breast tumorigenesis (13, 18–22). Here, we will focus on the molecular mechanisms by which obesity impact breast cancer pathogenesis. Particularly, we will outline the role of adiponectin in this type of cancer discussing epidemiological, *in vivo* and *in vitro* studies.

### Obesity and Breast Cancer

#### Epidemiology

In 2018 GLOBOCAN reported that worldwide breast cancer is the most diagnosed malignancy (2.1 million new cases) and represents the second cause of death in women (626,679 death accounting for 13% of cancer-related death). Several studies have reported the association between obesity, increased BMI and risk of breast cancer recurrence and mortality, showing important differences across menopausal status and disease subtypes. Nevertheless, in obese pre-menopausal women the reported data are still uncertain and controversial (23). It is fully demonstrated that obese post-menopausal women have a higher chance of developing breast cancer with a more advanced disease (larger tumor size, lymph-node positivity, regional/distance stage after diagnosis) compared with their normal counterpart (24). The correlation between breast cancer risk and obesity differs based on ER expression and menopausal status. Indeed, the development of estrogen receptor (ER) and progesterone receptor (PR)-positive breast cancer (24–28) markedly occurs in post-menopausal women, while the incidence of ER-negative tumors is more frequent in pre-menopausal patients (9). Besides, obese condition is considered a risk factor since overweight survivor breast cancer patients may develop a second primary malignancy, such as endometrial or colorectal neoplasia (29). Excess body weight may also negatively interfere in early diagnosis, therapeutic management, standard oncologic intervention and care of patients. In fact, in the obese population medical imaging and image-guided may be difficult to accomplish, while after surgical intervention probably may experience medical complications (30–34) and worst clinical outcome due to the dose-limited absorption of the conventional chemotherapy drugs (35, 36). Epidemiological studies well-described the role of obesity in impacting breast cancer treatment and prognosis but the biological mechanisms that link these pathological conditions are complex to elucidate. It has been demonstrated that the hyperactivation of Insulin/IGF-1 pathway due to “diabesity” condition (obesity concomitant with insulin resistance, hyperinsulinemia and/or hyperglycemia) mediates breast cancer progression (37–39). Obesity is also related to elevate circulating estrogen levels, as a result of increased aromatase activity in adipose tissue, chronically blunted inflammatory status and deregulated adipokine secretion, that bridge excess body weight condition and breast cancer.

#### Breast Cancer, Tumor Microenvironment and Adipose Tissue

Breast cancer is an intricate “rogue” organ wherein cancer cells surrounded by extracellular matrix and stromal cells create a complex tumor microenvironment (TME). Tumor cells and vital components of TME, such as fibroblasts and myofibroblasts, neuroendocrine, adipose, immune, inflammatory, and endothelial cells, interact *via* complex and dynamic network (40). Adipocytes are key components in the stroma of breast carcinoma. Indeed, adipose tissue is now recognized as a bioactive endocrine organ. Fletcher et al. demonstrated profound changes in the proteomic profile between the conditioned media obtained from human adipose tissue explants of breast tumors (hATT-CMs) compared to human adipose tissue explants of normal breast tissue (hATN-CMs). Most of the proteins differentially expressed were involved in important biological processes, including immune response and metabolism, and the treatment with hATT-CMs induces proliferation, adhesion and migration of breast cancer cells (41–43). In obese condition, adipocytes are hypertrophic, due to the increased accumulation of intracellular triglyceride stores caused by the energy imbalance, and hyperplastic, since their number augments in this pathology (44). Obese patients showed an excess of pathological and dysfunctional adipose tissue that leads to an enrichment of several biologically active factors (hormones, lipid metabolites, inflammatory cytokines, and adipokines) in the TME (45, 46). Hypertrophic and hyperplastic adipocytes through their metabolic substrates, such as both saturated and unsaturated fatty acids (FAs) and matrix metalloproteinase (MMPs), regulate breast cancer biology. FAs are stored as lipid droplet in the adipose tissue and their release, induced by high-fat diet and obese status, may contribute to angiogenesis and inflammation, key steps of tumor development and progression (47). Mostly, FAs can activate toll-like receptor 4 and NF-kB/TNF-α signaling. This contributes to create a low chronic inflammatory status of TME responsible for the recruitment of macrophages that encircle damaged, dying or dead adipocytes forming a crown-like structure (CLS) in adipose stromal breast tissue. Macrophage CLSs negatively influence breast cancer recurrence and survival (48–50). Adipocytes also through the direct release of several cytokines (IL-6, IL8, IFN_⋎_-inducible protein 10) and chemokines (CCL2 and CCL5) and the secretion of FAs, MMP-9, MMP-11, and MMP-13, may induce chronic inflammation, tumor initiation and metastatic burden in breast cancers. Moreover, adipocytes produce molecules, defined as “released hormones,” including estrogens and adipokines. In post-menopausal obese patients, the elevated level of estrogens, produced by aromatase highly expressed in adipocytes, activates ERα, promoting breast cancer cell growth and progression (47). Furthermore, changes in adipokines profile, due to the altered fat distribution and function, is one of the main features in obese state. Particularly, obese patients showed elevated serum leptin levels while adiponectin concentrations are decreased. It has been fully elucidated the role of leptin in breast cancer biology. Several studies reported that this adipokine secreted by adipocytes, tumor cells and cancer associated fibroblasts, induces the activation of several signaling pathways (i.e., JAK2/STAT3, MAPK, and PI3K/Akt) involved in the control of cell proliferation, differentiation, survival, migration, and invasion (51). Different epidemiological studies strongly support the inverse correlation between adiponectin and breast cancer, attributing to this adipokine a protective role. For instance, new evidences addressed its mechanistic involvement in breast tumor progression when present at low circulating levels (52–54).

## Adiponectin and Its Functions

**Figure 1:**
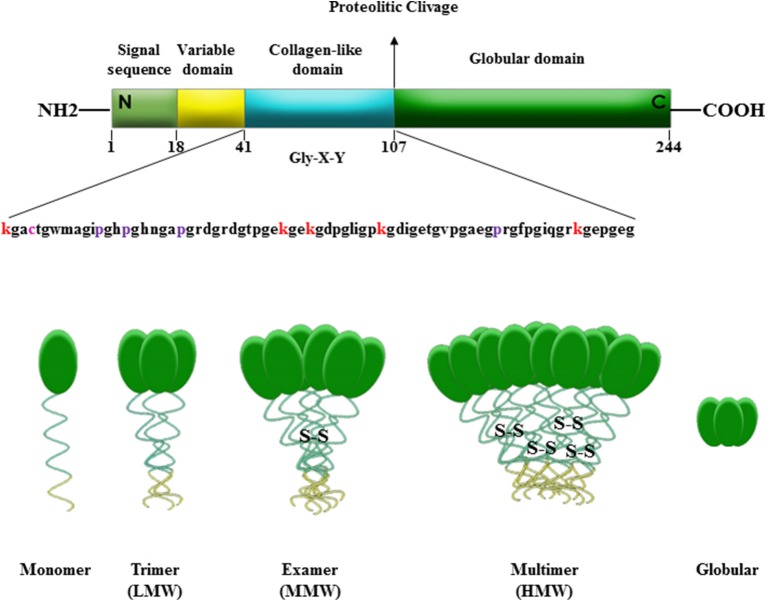
Domains and molecular structure of adiponectin. Adiponectin is a 244 amino acid protein mainly synthetized by adipocytes as a single subunit including a N-terminal signal sequence, a variable region, a collagen-like domain and a globular domain in C-terminus. Adiponectin before secretion undergoes to oligomerization to form trimers (LMW), hexamers (MMW), and multimers (HMW). Adiponectin is also present in plasma in a globular isoform (gAd) generated by a proteolytic cleavage at amino acid 110 of the full-length protein (fAd).

Adiponectin is mainly secreted by adipose tissue and in limited quantities by fat brown, salivary gland, cardiac tissue, cerebrospinal fluid, bone marrow (55–58). Adiponectin is synthetized as a single subunit with a primary sequence of 244 aminoacids divided into four domains: at N-terminus a signal peptide, followed by a short hyper-variable region, a collagenous region containing 22 Gly-X-Y repeats, and at C-terminus a globular domain that interacts with the receptors (39, 59–62). Once produced, the monomeric form of adiponectin undergoes to post-transcriptional modifications that allow the formation of disulfide-linked oligomers composed of trimers (Low Molecular Weight, LMW), hexamers (Medium Molecular Weight, MMW), and multimers (High Molecular Weight, HMW), detectable in the circulation (63–66). Furthermore, this adipokine exists in human plasma in a globular form (gAd), consisting only of the C-terminus domain, produced by a proteolytic cleavage of the full-length protein (fAd) at aminoacid 110 (60, 67) (Figure 1). Adiponectin exerts different biological functions, such as regulation of glucose uptake and insulin sensitivity, and stimulation of fatty acid oxidation (68–72). Moreover, adiponectin decreases the pro-inflammatory cytokines production (TNF-α and IL-6), prevents monocytes migration, affecting vascular endothelium, and mediates anti-atherogenic actions through the inhibition of subendothelial cholesterol accumulation (73–75). The response to adiponectin is related to the structural heterogeneity of serum adipokine isoforms and to the target tissues (70). Indeed, adiponectin activates two main seven-transmembrane receptors, Adiponectin Receptor 1 (AdipoR1) and 2 (AdipoR2). They are detected in almost every tissue, but generally with a different expression ratio and affinity for the adipokine oligomers (55, 62, 76, 77). Physiologically, adiponectin contributes to the 0.05% of total proteins present in the systemic circulation, with a concentration ranging from 3 to 30 μg/ml, depending on hormonal, inflammatory, pharmacological and dietary factors (70, 78, 79). Adiponectin plasma levels are reduced in obese compared to normal-weight subjects, negatively correlating with BMI (39, 80). The mechanisms responsible for this down-regulation have not yet been fully elucidated. It has been formulated several hypotheses, some of which are related to the chronic low inflammatory state of adipose tissue and to the adipocytes dysfunction that characterize obesity condition. Particularly, it has been speculated that the hypoadiponectinemia in obesity may be due to the increased production of pro-inflammatory cytokines, such as TNF-α and IL-6, or to a negative feedback of the adipokine on its own production and that of its receptors (39, 81, 82). Low adiponectin levels have also been linked to an increased risk of developing type 2 diabetes, metabolic syndrome, insulin resistance, hypertension, cardiovascular disease, and different malignancies, including breast cancer (59).

### Adiponectin and Breast Cancer

Pre-clinical studies with different experimental approached highlight the role of adiponectin on angiogenesis along breast tumorigenesis (83, 84). Using the transgenic mouse mammary tumor virus-polyoma middle tumor-antigen (MMTVPyV-mT) model of mammary cancer, it has been reported that adiponectin acts as a pro-angiogenic factor contributing to tumor vascularization and consequently to tumor growth and progression. Denzel et al. introducing adiponectin null mutation in MMTVPyV-mT (APN-KO) mice, a model closely related to human disease, found a delay in tumor onset, reduction in tumor growth kinetics and increased survival over wild type (WT) mice used as controls (83). Accordingly, another group reported that the chronic adiponectin depletion, after the use of an anti-mouse adiponectin monoclonal antibody, showed consistent and similar results obtained in APN-KO mice. On the other hand, a direct involvement of this adipokine in the angiogenic processes raised by the evidences that mice exposed to a series of adiponectin injection showed increased mRNA levels of the vascular epidermal growth factor (VEGF) release, well-known angiogenic factors, involved in blood vessel formation (84). Many epidemiological studied confirmed a positive correlation between hypoadiponectinemia and obesity-related breast cancer. Several case-control studies associated low circulating levels of adiponectin with an increased breast cancer risk and the development of a more aggressive phenotype in post-menopausal women, regardless of BMI, leptin, and IGF-I levels (85–92). Interestingly, this relationship has been observed prevalently in ER/PR-negative cancer (93). Furthermore, Oh et al. reported an inverse association between ER/PR-negative breast cancer recurrence and reduced serum adiponectin levels (93). This may be due to a cross-talk between adipose tissue-derived hormones, estrogens, progesterone and insulin signaling pathway that influenced adiponectin secretion (70). Nevertheless, a recent meta-analysis highlighted an increased incidence of intraepithelial breast tumor development and a higher risk of invasiveness in pre-menopausal women (94). Particularly, low adiponectin levels may favor tumor growth and progression, promoting fatty acid and protein synthesis or supporting the enhanced production of insulin, IGF-1, and pro-inflammatory cytokines (70). However, accumulating data demonstrated that the role of adiponectin in breast carcinogenesis, especially in post-menopausal women, could be closely dependent on cell types (60). Specifically, *in vitro* and *in vivo* studies evidenced a dichotomic effect on breast tumor growth and progression in relationship to ERα status (52–54, 95).

### Adiponectin and ERα-Negative Breast Cancer

Due to the importance of adiponectin in the regulation of breast cancer cell growth and proliferation, many investigations have attempted to elucidate the role of adiponectin accordingly to ERα expression. Several experimental models have documented the anti-proliferative and pro-apoptotic effects exerted by adiponectin in ERα-negative breast cancer cells (53, 96–102). Particularly, many evidences proved that even low adiponectin concentrations counteract breast carcinogenesis mediating the increase of Bax, p21, p53 expression and inducing cell cycle arrest, at G0/G1 phase, through the down-regulation of cyclin D1 (CD1) (54, 103). Moreover, adiponectin is able to up-regulate phosphorylation of AMPK, promoting TSC1/TSC2 complex formation, which in turn inhibits mTOR activation, and thus ERα-negative cell growth (52). Further investigations demonstrated the adiponectin negative action on lamellopodia formation, preventing cell migration and invasion (98, 104, 105). Additionally, a very recent RNA sequencing analysis (RNA-seq) highlighted the down-regulation of genes involved in G1/S and G2/M transition of cell cycle, including CD1, cyclin A2 and cyclin-dependent kinase 1, and CKAP2 and RRM1, implicated in the microtubule assembly and DNA replication during S phase, in adiponectin-treated MDA-MB-231 cells (52, 106–108). From the same analysis emerged the increased expression of genes involved in the cell cycle inhibition, such as *TGFBR1, IGFBP3*, and *MAGED1*, and genes sustaining apoptosis and cell death, among which *FAS, BIK, BID*, and caspases (52, 109–111).

### Adiponectin and ERα-Positive Breast Cancer

Several authors reported a protective role of adiponectin in ERα-positive breast cancer cells (86, 100, 105, 112–116), but divergent actions of the adipokine are emerging on this specific breast cancer subtype (52–54, 117, 118). Some studies described the inhibitory action of adiponectin on breast cancer cell growth by the up-regulation of p53, p21 and Bax and/or the reduced expression of c-myc, cyclin D, and Bcl-2 levels (78, 119, 120). Furthermore, it has been reported that full-length adiponectin induces apoptosis and cell death, promoting cytotoxic autophagy through the modulation of the LKB1-AMPK-ULK axis in breast cancer cells (121). Thus, based on the latter findings it is reasonable to speculate that prospectively the combined treatment of adiponectin with current chemotherapy drugs may reduce their effective dose lowering the side effects (121).

Recently, many evidences sustained the existence of a direct cross talk between adiponectin and ER signaling in breast cancer cells. In this concern, Pfeiler et al. demonstrated that estradiol (E_2_) treatment led to a down-regulation of AdipoR1 expression (117). Moreover, they showed that full-length adiponectin enhanced the E_2_-induced proliferation rate in ERα-positive MCF-7 breast cancer cells (117). Thus, the authors reported that the cross-talk between E_2_ and adiponectin affects breast cancer growth, highlighting an opposite action of adiponectin to that one generally assumed on human breast cancer cell behavior (117).

**Figure 2:**
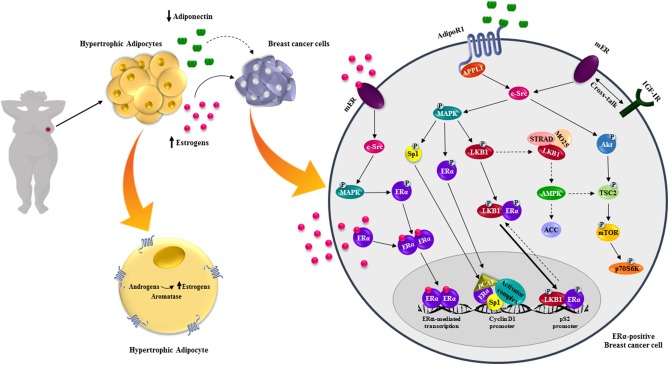
Effects of adiponectin on ERα-positive breast cancer cells. Obesity is characterized by hypertrophic adipocytes, which release high levels of estrogens, due to the increased aromatase activity, and low adiponectin concentrations. Estrogens bind and stimulate estrogen receptors action at both genomic and non-genomic levels. In ERα-positive breast cancer cells low adiponectin levels activate different intracellular signaling pathways promoting breast tumor growth.

Several reports described that adiponectin contributes to breast tumor evolution stimulating growth and migration of ERα-positive cells. Particularly, recent evidences elucidated several potential mechanisms through which globular adiponectin acts as a stimulatory factor in ERα-positive breast cancer (52–54). Mauro et al. demonstrated a dichotomic effect of adiponectin in ERα-positive and ERα-negative cells (53). They showed that adiponectin at 1 and 5 μg/ml, mimicking circulating levels in obese patients, exerted opposite effects between the two cell models. Adiponectin increased proliferation and cell growth in ERα-positive cells and these pro-tumor properties were completely abrogated either by ERα pharmacological inhibition (ICI 182,780), or by silencing its expression. Mechanistically, they found that adiponectin impacts ERα function at both non-genomic and genomic levels. Adiponectin treatment in MCF-7 cells increased the complex interaction between AdipoR1/APPL1 and mER/IGF1R/c-Src that leads to MAPK activation, sustaining cell growth. Moreover, through MAPK signaling, adiponectin was also able to trans-activates ERα, as demonstrated by its ability to increase XETL luciferase activity and the expression of classical ERα-responsive genes (pS2, Cathepsin D) (53). In summary, they proposed that ERα expression negatively interferes with the anti-proliferative effect mediated by adiponectin on breast cancer cell growth (53). Subsequently, *in vivo* experiments demonstrated an increased breast tumor growth in mice receiving MCF-7 cells pre-treated with adiponectin, concomitant with a marked increased expression of Ki67. All these finding were corroborated by experimental evidences reporting an up-regulation of CD1 expression in adiponectin-treated ERα-positive cells (54). Recently, it has been demonstrated that, upon adiponectin exposure, ERα/LKB1 interaction increased both in the cytosol and in the nucleus. All this contribute to impair LKB1 capability to activate AMPK and in such way weakening its consolidate role as onco-suppressor (52). Moreover, adiponectin enhanced Akt-induced TSC2 phosphorylation in MCF-7 cells, enabling mTOR to activate p70S6K. Notably, the absence of ERα, using a specific siRNA or the ICI 182,780, totally abrogated the adiponectin-mediated effects on LKB1/AMPK/mTOR signaling pathway (52). These data demonstrated the importance of ERα in regulating adiponectin actions in breast cancer cells (52). Furthermore, adiponectin increased ERα transcriptional activity only in the presence of LKB1, suggesting that LKB1 may be recruited as ERα co-activator (Figure 2). Finally, it has been shown that adiponectin exerts its effects in ERα-positive breast cancer cells in relation to its concentration. Indeed, 5 μg/ml adiponectin, simulating obese condition, induced cell proliferation and tumor growth, which was no longer noticeable at 30 μg/ml, corresponding to normal weight (52). RNA-seq fully elucidated how adiponectin doses (5 and 30 μg/ml) differentially influenced several important signaling pathways, modulating the expression of genes that control cell proliferation, cell cycle progression, apoptosis, cell death, necrosis and fatty acid metabolism. It has been reported that ERα expression switched the energy balance of breast cancer upon adiponectin exposure leading to a lipogenic more aggressive phenotype (52). Thus, these findings revealed a novel role of adiponectin in ERα-positive breast cancer, contributing to elucidate its controversial and previously reported dichotomic effects in this tumor setting. Adiponectin/AMPK axis also influences the circulating estrogens bioavailability through modulating aromatase activity in the breast. Brown et al. demonstrated that AMPK could phosphorylate CRTC2, one of the CREB-regulated transcription co-activators, blocking its nuclear translocation. At low adiponectin concentrations, not adequate to fuel AMPK activation, the non-phosphorylated CRCT2 binds CREB inducing transcription of target genes, including aromatase, in breast adipose tissues, contributing to tumor cell proliferation. Notably, this is a reasonable aspect linking low adiponectin levels with breast cancer growth and progression in obesity (122).

However, Swami et al. investigated the beneficial effects of dietary vitamin D supplementation on the enhanced estrogen synthesis in the breast microenvironment during obesity (123). Particularly, they observed, concomitantly with an enhanced AdipoR1 and adiponectin mRNA expression, a decrease of aromatase levels in mammary tumors of obese mice (123). Moreover, the authors evidenced that vitamin D could directly reduce aromatase synthesis by increasing the expression of LKB1, which drives AMPK phosphorylation in breast cancer cells. Overall, these data suggest that vitamin D may counteract the obesity-induced breast cancer growth, increasing adiponectin signaling and reducing local estrogen synthesis (123).

Since estrogens may influence adiponectin levels, it is worth to evaluate the possible link existing between adiponectin serum concentration and aromatase enzymatic activity in women with breast cancer. Recent preliminary clinical data evidenced that, in a small cohort of post-menopausal patients diagnosed with ERα-positive breast cancer and receiving aromatase inhibitors as adjuvant hormonal therapy, a short-term estrogen depletion did not affect adiponectin serum levels. Anyway, the limited clinical data available indicate that further efforts will be needed to provide translational relevance to these observations (124).

## Conclusions

Breast carcinoma is a complex and heterogeneous disease, whose development and progression depend not only on genetic predispositions. Indeed, it has been well-demonstrated that overweight and obese status deeply impact both breast cancer risk and behavior of the disease. An altered adipokine secretion, wherein adiponectin plasma levels dramatically decreased, mostly characterizes the obesity-related pathological expansion of adipose tissue. Although adiponectin is a well-recognized anticancer agent especially in breast cancer, recent studies suggested that the low adiponectin concentrations could amplify ER signaling, contributing to breast tumor development and progression. This led us to rethink adiponectin role in this neoplasia as a potential therapeutic tools. Prospectively, all new therapeutic strategies aimed to potentiate adiponectin actions (i.e., increasing its circulating levels or the binding to its own receptor) should be carefully assessed separately in ERα-positive and ERα-negative breast cancers.

## Author Contributions

GN and LG reviewed the literature, prepared the manuscript, wrote, edited, and prepared the figures. SC, LM, and SA revised and provided the critical consideration for the manuscript, design, and editing. All authors have read and approved the final version of this manuscript agreed to be accountable for all aspects of the work and consent for publication.

### Conflict of Interest

The authors declare that the research was conducted in the absence of any commercial or financial relationships that could be construed as a potential conflict of interest.

## Abbreviations

BMI: Body Mass Index
ER: Estrogen Receptor
PR: Progesterone Receptor
IGF-1: Insulin-like Growth Factor
TME: Tumor Microenvironment
hATT-CMs: human Adipose Tissue explants of breast Tumors
hATN-CMs: human Adipose Tissue explants of Normal breast tissue
FAs: Fatty Acids
MMPs: Matrix Metalloproteinase
TNF-α: Tumor Necrosis Factor α
CLS: Crown-Like Structure
IL-6: Interleukin-6
IL-8: Interleukin-8
CCL2: C-C Motif Chemokine Ligand 2
CCL5: C-C Motif Chemokine Ligand 5
JAK2: Janus Kinase 2
STAT3: Signal Transducer and Activator of Transcription 3
MAPK: Mitogen-Activated Protein Kinase
PI3K: Phosphatidylinositol 3-Kinases
LMW: Low Molecular Weight
MMW: Medium Molecular Weight
HMW: High Molecular Weight
gAd: globular Adiponectin
fAd: full-length Adiponectin
AdipoR1: Adiponectin Receptor 1
AdipoR2: Adiponectin Receptor 2
CD1: Cyclin D1
AMPK: 5′ Adenosine Monophosphate-activated Protein Kinase
TSC1: Tuberous Sclerosis 1
TSC2: Tuberous Sclerosis 2
mTOR: mammalian Target Of Rapamycin
RNA-seq: RNA sequencing
MMTVPyV-mT: Mouse Mammary Tumor Virus-Polyoma middle Tumor-antigen
WT: Wild Type
VEGF: Vascular Epidermal Growth Factor
E_2_: Estradiol
APPL1: Adaptor protein, Phosphotyrosine interacting with PH domain and Leucine zipper 1
LKB1: Liver Kinase B1
CRTC2: CREB Regulated Transcription Coactivator 2.
